# Plant Extracts Downregulating the JAK/STAT Signaling Pathway as a Potential Tool for Psoriasis Management: A Review

**DOI:** 10.3390/ph19050699

**Published:** 2026-04-29

**Authors:** Maria Rosaria Perri, Giulia Conforti, Eugenio Provenzano, Maria Itria Giancotta, Claudia-Crina Toma, Filomena Conforti, Giancarlo Statti

**Affiliations:** 1 Department of Pharmacy, Health and Nutritional Sciences, University of Calabria, 87036 Rende, Italy; mariarosaria.perri@unical.it (M.R.P.); filomena.conforti@unical.it (F.C.); 2 Ordine dei Medici-Chirurghi e degli Odontoiatri, Via Suor Elena Aiello 23, 87100 Cosenza, Italy; confortigiulia98@gmail.com; 3 UOC di Dermatologia AO Cosenza, 87100 Cosenza, Italy; e.provenzano@aocs.it; 4 Allergologia ASP Cosenza, 87100 Cosenza, Italy; mariaitriagiancotta@libero.it; 5 Pharmacognosy Department, Faculty of Pharmacy, Vasile Goldis Western University of Arad, 310045 Arad, Romania

**Keywords:** plant extracts, psoriasis, inflammatory skin diseases, JAK/STAT Signaling Pathway, cytokines, phytochemicals

## Abstract

**Background/Objectives:** Among the Inflammatory Skin Diseases (ISDs), psoriasis represents a recalcitrant and disabling dermatological disorder associated with perpetuated inflammatory conditions. Recent findings showed that psoriasis pathogenesis is sustained by the interplay of different inflammatory Signaling Pathways, among which the JAK/STAT stands out. **Methods:** This review aims to investigate the state of the art and the most recent insights concerning the efficacy of plant extracts in psoriasis management via modulation of the JAK/STAT Signaling Pathway. **Results:** In vitro and in vivo studies revealed that several different plant species showed promising results in modulating the dysregulation of the JAK/STAT Signaling Pathway associated with psoriasis. Raw and enriched extracts and herbal remedy formulations were discussed. Then, present insights, future directions, and gaps of literature were addressed. **Conclusions:** Promising studies suggested that plant-based products, able to downregulate the JAK/STAT Signaling Pathway, could represent an effective tool for psoriasis management.

## 1. Introduction

### 1.1. An Overview of Inflammatory Skin Diseases (ISD)

The skin, as the largest and major exposed vital organ, exerts a wide range of functions, including body temperature regulation, protection against infections and preservation of inner organs. For all these reasons, guaranteeing the integrity of the skin barrier and ensuring the maintenance of skin health are currently necessary and strategic challenges [1,2,3]. ISDs represent a cluster of heterogeneous pathologies, commonly defined as T-cell-mediated disorders. ISDs are characterized by abnormal responses of immune cells to various stimuli (endogenous and/or exogenous) triggering chronic inflammatory processes. Genetic, infectious, immune system, lifestyle and environmental factors play a key role in the development of ISDs, whose clinical manifestations are erythema, scales and itching [4]. ISDs include psoriasis, eczema (or atopic dermatitis), hidradenitis suppurativa, systemic sclerosis (scleroderma), etc. [5]. Epidemiological data showed that ISDs affect more than 1/5 of the entire human population [6]. This cluster of disorders is currently considered a leading cause of disability all over the world, often associated with comorbidities including chronic kidney diseases, cerebral-arteriosclerosis, cardiomyopathy, abnormal fat metabolism, systemic amyloidosis, etc. [7,8,9]. Furthermore, a population-based study, conducted by Mann and colleagues (2025), indicated that children with ISD show increased risks to develop sleep psychological disorders such as hyper insomnia, sleep apnea, fatigue and depression [10].

### 1.2. The Involvement of the JAK/STAT Signaling Pathway in Psoriasis Pathogenesis

Among all the ISDs, psoriasis, affecting forty to sixty million people all over the world, is considered one of the most prevalent chronic, autoinflammatory, dermatological diseases, mainly sustained by innate immune mechanisms. Psoriasis is widely characterized by erythematous, scaly plaques and nail dystrophy [11,12]. It is a multifactorial disorder occurring at any time, whose prevalence usually increases proportionally to advancing age [13,14]. People affected by psoriasis often show increased risks of developing comorbidities and complications such as obesity, diabetes mellitus, cardiovascular risk, inflammatory bowel disease and joint pain [15]. Recent findings also suggest a non-negligible correlation between psoriasis and incidence of stroke, mental disorders and cancer [16,17]. Several therapeutic approaches for psoriasis treatment are available, but unfortunately, none can be considered resolutive. Psoriasis etiology remains generally unclear: genetic predisposition, as 63 psoriasis susceptibility loci were identified, and environmental factors are considered the main triggering factors [18,19]. From a pathogenic point of view, psoriatic lesions occur after hyperproliferation and differentiation of epidermal keratinocytes triggered by Interleukin (IL) (IL-23, IL-17) pathway mediators. T helper 17 cells (Th17) lymphocytes, dendritic cells and neutrophils are also considered key players in psoriasis pathogenesis [12,20]. Furthermore, epigenetic and metabolic reprogramming seems to exert a key role in psoriasis pathogenesis [21]. Recent findings suggest that the Janus Kinase Signal Transducer and Activator of Transcription (JAK/STAT) Signaling Pathway play a pivotal role in several inflammatory immune responses and, overall, in the pathogenesis of many different disorders [22,23]. The reason can be retrieved in the fact that cytokines involved in these pathologies use the JAK/STAT Signaling Pathway as a transduction signal. In fact, when circulating cytokines, like IL-6 or interferon (IFN), couple with the extracellular domain of the receptor, a conformational change occurs and two JAKs are recruited. JAKs phosphorylate downstream signaling proteins, including STATs, which undergo phosphorylation and dimerization. Dimerized STATs proteins can translocate into the nucleus and modulate gene transcription. Moreover, in psoriasis, where the IL-17/IL-23 axis plays a crucial role, the inhibition of the JAK/STAT Signaling Pathway is gaining interesting clinical significance [24,25]. This pathway was described for the first time about 30 years ago and, at present, is considered a therapeutic target for rheumatoid arthritis, psoriatic arthritis, juvenile arthritis, ankylosing spondyloarthritis, ulcerative colitis, atopic dermatitis, alopecia areata, graft-versus-host disease, myeloproliferative neoplasms, Coronavirus disease 2019 (COVID-19), vitiligo and psoriasis [26]. Psoriasis, in particular, is characterized by the dysregulation of immune response and a persistent inflammatory process, sustained by increased circulation of pro-inflammatory cytokines like IL-9 and IL-22. Specifically, abnormal levels of circulating cytokines are responsible for both JAK/STAT Signaling Pathway activation and abnormal cell proliferation in involved tissues. In particular, the aberrant activation of STAT3 seems to be implicated in highly induced Th17 differentiation, increased IL-22 production and the occurrence of spontaneous psoriatic lesions in mouse models [12]. STAT3 hyperactivation is correlated to the overall onset and maintenance of the disease, being considered a modulator of inflammatory and immune response [27]. A pilot study conducted by Du and colleagues (2020), in which skin biopsies of 55 psoriatic patients were analyzed, revealed that STAT1, STAT3, Suppressor of Cytokine Signaling 1 (SOCS1), Suppressor of Cytokine Signaling 3 (SOCS3) and Vascular Endothelial Growth Factor (VEGF) were significantly higher in psoriatic lesions when compared to the control group [28]. The JAK/STAT Signaling Pathway can be targeted at various levels, with different strategies and several approaches, most of them probably still unknown, which represent the extraordinary pharmacological relevance of this Signaling Pathway [24].

### 1.3. Therapeutic Approaches Targeting the JAK/STAT Signaling Pathway

Currently, a new class of small-molecules drugs, the Janus Kinase inhibitors (JAKis), able to properly target the JAK/STAT Signaling Pathway is gaining even more attention and accreditation as a tool for the management of immune-mediated inflammatory conditions, including moderate-to-severe psoriasis [29,30]. Thanks to their small molecular size (<500 kDa), JAK inhibitors can pass cell membranes and act at the intra-cellular level. They can be administered in oral, transcutaneous and transmucosal way, and they do not require any metabolic activation. In particular, Tofacitininb, Upadacitinib, and Deucravacitinib, the only three JAK inhibitors drugs approved by both the Food and Drug Administration (FDA) and European Medicine Agency (EMA), showed efficacy and a good safety profile, even if no long-term use data are available yet. Other JAK inhibitors are instead correlated to an increased risk of contracting herpes zoster and infections and the delivery of overall adverse events [11]. The validated mechanism of action involves the downregulation of the evolutionarily conserved JAK/STAT Signaling Pathway implicated, through type I and II receptors, in cytokines signaling. In 2023, EMA recommended the use of JAKis only in patients over 65 and who are smokers, considered refractory to all the other treatments. Furthermore, cautions and lower doses are necessary in case of cancer, thromboembolisms and cardiovascular risk [11]. Ethnobotanical studies revealed that different plants, over time, have been empirically and traditionally used for psoriasis management [31]. According to Nguyen (2022), almost 50% and 60% of people affected by psoriasis in Europe and Asia, respectively, used natural medicine products during psoriasis treatment, considering this approach both safe and cost-effective [32]. In this context, plant-derived extracts, representing a source of effective bioactive compounds including flavonoids, phenolic acids, tannins and sesquiterpenes, could be considered potential and effective dermatological phytotherapeutics [33]. This work aimed to investigate current findings about the growing body of evidence concerning the potential role of plant extracts in the treatment of psoriasis via modulation of the JAK/STAT Signaling Pathway. Plant species, in fact, have a twofold advantage: on the one hand, they are responsible for fewer and milder side effects than commonly used drugs; on the other hand, they can effectively attenuate the chronic inflammatory process underlying psoriasis. By bringing together all the latest updated knowledge in the field of this subject, this work aims to connect evidence about the potential efficacy of plant extracts.

## 2. Plant Extracts Targeting the JAK/STAT Signaling Pathway in In Vitro and In Vivo Psoriatic Models

**Figure 1:**
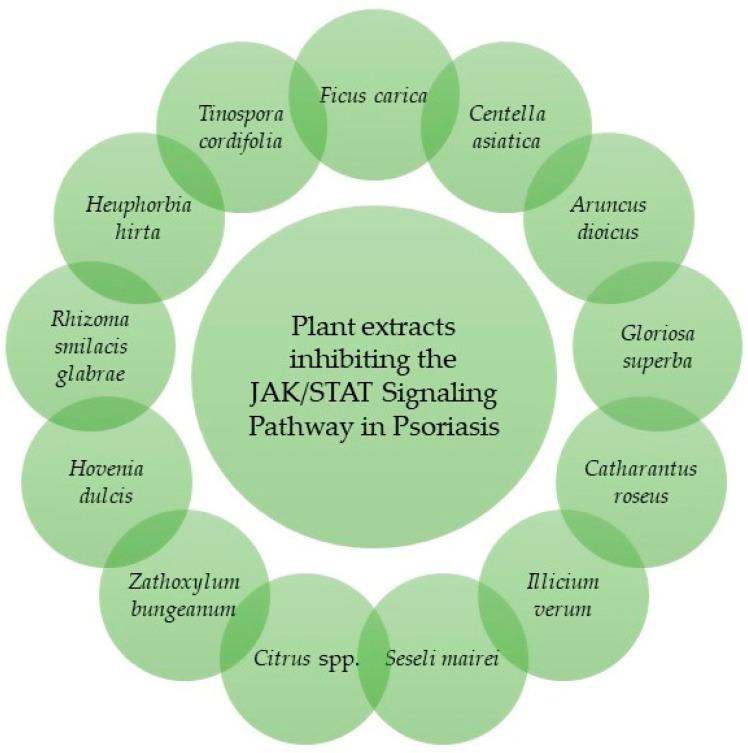
Plant extracts inhibiting the JAK/STAT Signaling Pathway in psoriasis.

Thanks to their efficacy, safety and low toxicity, plant-derived extracts represent a promising approach for the management of psoriasis [34]. Products able to modulate the JAK/STAT Signaling Pathway in psoriatic models are discussed below (Figure 1, Table 1).

### 2.1. Ficus carica L.

*Ficus carica* L. was traditionally used for a wide range of beneficial properties and protective effects on gastrointestinal, urinary and respiratory tracts. Over the years, its main bioactive compounds, such as flavonoids and coumarins, were identified. Lee and colleagues (2023) [35] investigated the in vitro anti-inflammatory potential and the in vivo ability to ameliorate psoriatic conditions of the *F. carica* fruit extract. Methanolic extract, obtained through ultrasonication in concentrations ranging from 50 to 200 µg/mL, was investigated in RAW264.7 murine macrophages, previously stimulated with Lipopolysaccharide (LPS). *F. carica* fruit extract significantly reduced Nitric Oxide (NO), Inducible Nitric Oxide Synthase (iNOS) expression, and both JAK1 and STAT3 phosphorylated proteins. Moreover, the anti-inflammatory effects of the extract were tested in a six-week-old male BALB/c psoriasis-like mouse model: data showed that erythema, scales and dorsal skin thickness scores, considering typical manifestations of psoriasis, were significantly decreased. Additionally, the animal group treated with the highest dose of extract (10 mg/mL) showed amelioration signs similar to that observed in the control group, treated with dexamethasone. The spleen was also investigated: as a result, its weight appeared significantly reduced in mice treated with *F. carica* fruit extract high doses. Interesting results of this study also revealed that, while Nuclear Factor-kappa B (NF-kB) and Mitogen-activated protein kinase (MAPK), traditionally recognized as inflammatory pathway, were not modulated, the JAK/STAT Signaling Pathway underwent significant downregulation by *F. carica* extract, suggesting a direct involvement in psoriasis pathogenesis [35].

### 2.2. Centella asiatica (L.) Urban

*Centella asiatica* (L.) Urban (Umbelliferae), widely spread in China as herbal tea, was traditionally used for the treatment of cardiovascular, respiratory, digestive and dermatological disorders. In reference to the last mentioned effect, the efficacy of *C. asiatica* extract in counteracting skin inflammation, skin ulcers, atrophic lichen sclerosus, keloids and scleroderma, acne, burns, atopic dermatitis and wound healing was already demonstrated. Lin and colleagues (2023) [36] investigated the role of *C. asiatica* extract on inflammatory dermatosis. Plant material was subjected to consecutive processes of macerations and ultrasonication, allowing them to achieve *n*-hexane (CAH), ethyl acetate (CAE), *n*-butanol (CAB) and aqueous (CAA) extracts, respectively. *C. asiatica* extract’s anti-inflammatory potential was investigated by treating HaCaT cells, previously stimulated with LPS (20 µg/mL). Data showed that all the investigated samples inhibited mRNA levels of inflammation-related factors, with CAH and CAE being the most effective. In particular, CAH, at the final tested concentration of 25 µg/mL, inhibited the expression of C-C motif chemokine ligand 20 (CCL20), IL-6 and Tumor Necrosis Factor-alpha (TNF-α) by 100.4%, 51% and 124.3%, respectively. In parallel, CAE (25 µg/mL) reduced the expression of the same factors by 69.3%, 34.2% and 84.3%, respectively. CAH and CAE induced the expression of Aquaporin 3 (AQP3) and Filaggrin (FLG), two genes involved in the maintenance of the skin barrier function stability, when compared to cells treated with LPS only. Additionally, CAE (25 µg/mL) significantly downregulated the Toll-like Receptor 4/Nuclear Factor-kappa B (TLR4/NF-kB) pathway in a dose-dependent manner and the JAK/STAT3 cascade by suppressing the phosphorylation of JAK1 and STAT3 proteins at 50.4% and 73.9%, respectively. The dermatological efficacy of *C. asiatica* extract was also determined in vivo, in an Imiquimod (IMQ) 5% induced psoriasis-like skin inflammation mice model. CAE treatment at 40 mg/mL, for 7 days, reduced skin scale, blood scab and inhibited the release of inflammatory factors both in serum and skin lesions. In conclusion, through the inhibition of IL-6, responsible for JAK1 and STAT3 phosphorylation, abnormal keratinocytes proliferation, and T cells migration, *C. asiatica* extract resulted in effective in psoriatic models [36].

### 2.3. Aruncus dioicus var. kamtschaticus

*Aruncus dioicus* var. *kamtschaticus*, commonly known as “goat’s beard”, is a Chinese and Korean traditional medicine mainly used for its antioxidant, anti-inflammatory, antimicrobial and anticancer properties. Dorjsembe and colleagues (2022) [37] investigated its anti-inflammatory potential in a psoriasis model, both through in vitro and in vivo studies. The *A. dioicus* sample was obtained by extracting aerial parts in EtOH. The psoriasis-like model was induced in male BALB/c mice (7 weeks old) by applying 62.5 mg of IMQ cream daily (1 week). Then, animals were treated with 100 µL of *A. dioicus* extract at a low dose (1 mg/mouse) and high dose (2 mg/mouse), respectively, for another week. Dexamethasone was used as positive control. The application of *A. dioicus* extract at a high dose significantly inhibited erythema, infiltration, and scales, reversed phenotypical changes induced by IMQ and decreased Psoriasis Area and Severity Index (PASI) score, in a manner comparable to dexamethasone. Moreover, *A. dioicus* extract topical application decreased the migration of immune cells to skin lesion, by reducing Cluster of Differentiation 3-positive (CD3^+^) T cells and F4/80^+^ macrophages infiltration. The extract, both at low and high doses, suppressed the expression of pro-inflammatory cytokines (IL-1β, IL-23), C-X-C motif chemokine ligand (CXCL1, CXCL2, CXCL10), CCL20 and antimicrobial peptides S100 calcium binding protein A8 (S100A8) and S100 calcium binding protein A9 (S100A9). The efficacy of the extract was also evaluated in HaCaT cells stimulated with a cocktail of IL-17a and TNF-α. *A. dioicus* extract efficacy was tested at 25, 50 and 100 µg/mL, respectively, concentrations that did not affect cell viability. As a result, data showed that the extract attenuated the upregulation of IL-17a/TNF-α-induced markers including IL-1β, IL-23, CXCL1, CXCL2, CXCL10, CCL20, S100A8 and S100A9. Western blot analyses also revealed that *A. dioicus* extract treatment (25, 50, 100 µg/mL) reduced mRNA gene expression of psoriasis hallmarks, phosphorylation of JAK2/STAT3 and protein kinase B (Akt)/mammalian target of rapamycin (mTOR) Signaling Pathways in a dose-dependent manner. These findings suggested that prevention of psoriatic symptoms by *A. dioicus* extract could be attributable to the inhibition of the JAK/STAT Signaling Pathway [37].

### 2.4. Gloriosa superba L.

*Gloriosa superba* L. is a traditional herbal medicine mainly used, over the centuries, for the treatment of dermatological disorders but also employed for bruises, infertility and joint pain. Pattarachotanant and colleagues (2014) [38] investigated the anti-psoriatic potential of *G. superba* leaf and stem extracts obtained through EtOH maceration. HaCaT cells were stimulated with IFN-γ in order to induce the expression of Keratin 17 (K17), a protein contributing to psoriatic process. Then cells were treated with extract at concentrations ranging from 0.01 to 10 µg/mL. *G. superba* extract decreased K17 expression in a dose-dependent manner. Furthermore, Western blot analyses revealed that the investigated extract, at all the tested concentrations, significantly decreased phosphorylation of STAT3 proteins, a mechanism directly involved in K17 expression. [38].

### 2.5. Catharanthus roseus (L.) G.Don

*Catharanthus roseus* (L.) G.Don is a traditional medicine mainly used for menorrhagia, diabetes, hypertension and cancer. Pattarachotanant and colleagues (2014) [38] investigated the efficacy of *C. roseus* leaf and stem EtOH extract as a potential anti-psoriatic agent in a HaCaT cell model. Amounts of 10, 25, 50 and 100 µg/mL of the extract were added to cells previously treated with IFN-γ (1 ng/mL), in order to stimulate the expression of K17. As a result, Western blot analyses revealed that the extract decreased the expression of K17 in a dose-dependent manner and inhibited phosphorylation of STAT3 proteins. Possibly, the K17 inhibitory expression could be attributable to the downregulation of the JAK/STAT Signaling Pathway [38].

### 2.6. Illicium verum Hook.f.

*Illicum verum* (Illiciaceae), commonly known as “star anise”, is an ethnopharmacologically relevant plant traditionally used in Asia for skin inflammation, rheumatisms, asthma and bronchitis. *I. verum* dried fruits were extracted in EtOH 70%. Sung and colleagues (2013) [39] investigated the anti-inflammatory potential and the related mechanism of action of *I. verum* fruit extract (IVF) in human keratinocytes (HaCaT cell lines). IVF (25, 50 and 100 µg/mL) significantly decreased Intercellular Adhesion Molecule 1 (ICAM-1) mRNA expression in IFN-γ stimulated cells, without affecting cell viability. Furthermore, by investigating the JAK/STAT signaling activation, data revealed that IVE treatment inhibited IFN-γ-induced phosphorylation of JAK2 and STAT1 proteins in a dose-dependent manner. Surprisingly, IVE treatment (100 µg/mL) exerted 100% inhibition of JAK2 phosphorylation and increased SOCS1 expression, responsible for the downregulation of p-JAK2 and, consequently, of p-STAT1 [39].

### 2.7. Seseli mairei Wolff

*Seseli mairei* Wolff (Umbelliferae) is a plant species widely used as an antibacterial, anti-inflammatory, antiallergic and antipyretic agent. It was traditionally used for psoriasis and skin and immune diseases. Wang and colleagues (2023) [40] investigated both the in vitro and the in vivo biological potential of *S. mairei* extract (SME) obtained through heat reflux of roots in EtOH 95%. As regards the in vitro study, HaCaT cells were treated with SME in growing concentrations, which demonstrated a gradual reduction in cell density. The extract concentrations, which did not affect cell viability (ranging from 0.025 to 0.05 mg/mL), significantly reduced levels of the pleiotropic IL-6 cytokine in HaCaT cells previously stimulated with IL-17a. Then, the efficacy of the extract, both at high (300 mg/kg) and low dose (150 mg/kg) and after 6 days of oral administration, was tested in vivo in psoriasis-like dermatitis mice treated with IMQ. SME significantly reduced epidermal thickness, inflammatory cell infiltration and dermis hyperkeratosis. The spleen index of mice treated with SME was also significantly ameliorated when compared to the control group, suggesting a reduction in the overall systemic inflammatory response. Furthermore, levels of psoriasis-related cytokines were evaluated: data showed that SME treatment decreased the expression of IL-1β, IL-6, IL-8, IL-17A, IL-22 and IL-23 and increased the release of IL-10, as assessed by both immunohistochemistry analyses and ELISA test. CD4+IL-17A+ proportion significantly decreased in mice spleen treated with SME at a high dose, highlighting how the extract could be effective in inhibiting Th17 cells, considered responsible for harmful cytokines secretion in the psoriasis process. Then, Signaling Pathways involved in the psoriasis process were investigated through Western blot analyses. As a result, SME treatment decreased expression levels of NF-kB, STAT3 and JAK2 proteins, suggesting that the protective activity of the extract could occur via the above-mentioned Signaling Pathway inhibitions of which interplay is currently recognized as implicated in psoriasis pathogenesis [40].

### 2.8. Citrus spp. and Zanthoxylum bungeanum Maxim

Plants belonging to the Rutaceae family, including *Citrus medica* Linn. (CM), *Citrus aurantium* L. Cv. Daidai (CA), *Citrus medica* Linn. var. *sarcodactylis* (Noot.) Swingle (CMS), *Citrus sinensis* (L.) Osbeck (CS) and *Zanthoxylum bungeanum* Maxim (ZB), widely consumed as dietary supplements, are well recognized for their antioxidant and anti-inflammatory properties. Mengsa and colleagues (2022) [41] investigated and compared the biological properties of the EtOH 50% extracts obtained from these plant species. Total phenolic and flavonoid content, as well as antioxidant potential, were assessed. HaCaT cells were treated with H_2_O_2_ (400 µmol/L) and LPS (20 µg/mL) in order to simulate oxidative and inflammatory injury; cell viability was investigated through MTT assay by treating cells with extracts in concentrations ranging from 1 to 250 µg/mL. Then, Reactive Oxygen Species (ROS), Superoxide Dismutase (SOD) and Glutathione (GSH) were evaluated, resulting in amelioration after extract treatments. All the analyzed extracts, at the concentration of 50 µg/mL, inhibited p-JAK2 proteins by 51.78%, 36.26%, 61.94%, 40.90%, and 29.84% and p-STAT3 proteins by 100.83%, 88.69%, 44.50%, 51.71% and 53.04%, respectively. High-dose extracts also suppressed p-38 MAPK phosphorylation and increased AMP-activated protein kinase (AMPK) expression. All the extracts, both at 5 µg/mL and 50 µg/mL, significantly suppressed TNF-α, IL-6, IFN-γ, CCL20 and Matrix Metallopeptidase 1 (MMP1). Among all the tested extracts, *Z. bungeanum* showed the best activity, being able to both downregulate NF-kB and the JAK/STAT3 Signaling Pathway, ameliorating skin inflammation and hyperplasia, which suggests that it could be used as a promising anti-psoriatic agent [41].

### 2.9. Hovenia dulcis Thunb.

*Hovenia dulcis* Thunb. is a plant species traditionally used for liver disorders and alcohol poisoning. Kim and colleagues (2023) [42] investigated *H. dulcis* (HD) anti-inflammatory properties in vitro. Dried fruits of HD were extracted with MeOH through an ultrasonication process (50 °C). HD treatment, at concentrations of 6.25 and 25 µg/mL, prevented keratinocytes hyperproliferation and suppressed mRNA expression of IL-1α, IL-1β, CCL-20, CXCL-8 in HaCaT cells stimulated with TNF-α, without affecting cell viability. Moreover, while TNF-α significantly induced phosphorylation of STAT3 proteins, HD treatment reduced p-STAT3 in a dose-dependent manner. The NF-kB and STAT3 downregulation was mediated by MAPK modulation in TNF-α induced psoriatic keratinocyte models [42].

### 2.10. Rhizoma Smilacis Glabrae

*Rhizoma Smilacis Glabrae* (RSG) is a well-known immunosuppressant herb and a potential anti-rheumatic drug due to its high favorable tolerability profile. Tang and colleagues (2023) [43] investigated the anti-psoriatic properties of the RSG extract, obtained through heating and refluxing in a mixture of EtOH 75% and ethyl acetate. High-Performance Liquid Chromatography (HPLC) fingerprint analyses of the extract revealed 10 peaks, while physicochemical and spectroscopic data showed 49 compounds. BALB/c mice in an IMQ-induced psoriasis model were treated with low (23.3 mg/kg), medium (35.0 mg/kg) and high (46.6 mg/kg) doses of RSG. High-dose treated mice showed a significant reduction in erythema, edema and ulceration of the skin lesion. IL-17, IFN-γ and TNF-α cytokine expression as well as Retinoic acid-inducible gene I (Rig-1), GATA binding protein 3 (Gata3), mTOR, JAK, STAT and p-65 expression levels were evaluated: RSG downregulated Rig-1, Gata3, mTOR and JAK in a dose-dependent manner. RSG also suppressed Th17/Treg ratio without affecting cell viability in HaCaT cell models [43].

### 2.11. Euphorbia hirta L.

*Euphorbia hirta* L. (Euphorbiaceae) is a plant species traditionally used to treat respiratory disease, allergic asthma, gastrointestinal disorders, inflammation and malaria. Gil and colleagues (2022) [44] investigated the in vitro potential of EtOH 70% *E. hirta* leaf extract (EHE) for skin inflammatory diseases. HaCaT cells were pretreated with 60, 120 and 240 µg/mL of EHE, then stimulated with a cocktail of TNF-α/IFN-γ. EHE, at the tested doses of 120 and 240 µg/mL, significantly reduced mRNA expression levels of TNF-α, IL-6, CCL22 and CCL5. Moreover, EHE treatment downregulated Akt phosphorylation and periostin expression, a crucial marker of allergic reaction. In general, all EHE concentrations and, in particular, the two highest tested doses downregulated the c-jun N-terminal kinase (JNK)/MAPK pathway and inhibited phosphorylation of JAK2 proteins and STAT1/STAT3 residues of serine and tyrosine in TNFα/IFN-γ stimulated cells [44].

### 2.12. Tinospora cordifolia (Willd.) Hook.f. & Thomson

*Tinospora cordifolia* (Willd.) Hook.f. & Thomson, traditionally used in Ayurveda medicine, is widely employed for the treatment of inflammatory conditions, autoimmune disorders and cancer. Nandan and colleagues (2023) [45] investigated both the in vitro and the in vivo antipsoriatic effects of a commercial *T. cordifolia* (TC) aqueous extract. The chemical fingerprint, assessed by LC-MS analyses, revealed reticuline, tinocordiside, 20β-hydroxyecdysone, cordifolioside A, tinosporinone, jatrorrhizine and cordifolide as the main secondary metabolites. TC, at concentrations of 500, 1000, 1500 and 2000 µg/mL, was tested in CD4^+^ T cells: no toxicity was detected after 24 h of incubation, while the highest tested dose (2000 µg/mL) affected cell viability in a moderate manner after 96 h of exposure. Native mouse CD4^+^ T cells, treated with TC and cultured under Th17-polarizing conditions, affected IL-17 release in a dose-dependent manner at concentrations higher than 1500 µg/mL. At the same tested concentration, suppression of cytokines production and receptor Signaling Pathway via the JAK/STATSignaling Pathway, as well as neutrophil degranulation and TLR-3/TLR-4 modulation, were observed. Additionally, CD4^+^ T differentiated cells, treated with TC, showed a significant downregulation of FoxP3 expression, already at the lowest tested dose of 500 µg/mL [45].

### 2.13. Lavandula angustifolia Mill.

*Lavandula angustifolia* Mill. (Lamiaceae) is an aromatic plant with high ethnopharmacological relevance, well-known for the beneficial properties of its essential oils. Previous studies have already validated the effects of the topical application of lavender oil for psoriasis. Rosmarinic Acid (RA), a phytochemical mainly spread in plants from the Lamiaceae family, showed a plethora of biological properties including antioxidant, anti-inflammatory, anti-allergic, antifibrotic, anti-adipogenic, neuroprotective and photo-protective. Despite the interesting pharmacological properties, its production is disadvantageous, since the extraction yield is less than 1% of dry material. In this context, the beneficial properties of *L. angustifolia* calli culture (LA), able to produce RA 1.7-fold more than control, was investigated and the phytochemical quantification of RA was determined by HPLC. Data showed that cell suspension held a RA amount of 100.16 ± 7.01 mg/g of extract. Cell viability assay on HaCaT cells revealed that LA extract (20, 40 and 100 µg/mL) and RA (5, 10, 25 µM) did not affect cell viability. As a result, LA treatment significantly inhibited mRNA expression of genes connected to NF-kB signaling, STAT3, AKT and MAPK8 genes, suggesting a modulation of JAK/STAT, MAPK and P13K/AKT pathways. In parallel, RA treatment induced the same effects in a dose-dependent manner and in similar transcriptional changes, while exerting higher activity in downregulating IL-6 and CCL20 mRNA expression. In conclusion, LA extract, containing RA 10%, modulated the JAK2/STAT1 Signaling Pathway in psoriasis-like inflammation cell models [46].

### 2.14. Pinus massoniana Lamb.

*Pinus massoniana* Lamb. (Pinaceaea) shows high therapeutic relevance thanks to its anti-inflammatory, antihypertensive, antitumor, antioxidant and neuroprotective activities. In particular, pine pollen polysaccharides (composed of glucose and arabinose) and pine needles (rich in dihydroquercetin (DHQ), quercetin and catechin) gained even more attention over previous years due to their antitumor, immunomodulatory and cardioprotective properties. Xu and colleagues (2026) [47] investigated the anti-psoriatic mechanism of pine pollen extract (PPE) rich in DHQ. The extract was subjected to preliminary processes of defatting with petroleum ether, and then the obtained residue was extracted in EtOH 40%. Both qualitative and quantitative phytochemical analyses were conducted in Ultra-Performance Liquid Chromatography-Quadrupole-Orbitrap Mass Spectrometry (UPLC-Q-Orbitrap/MS) in order to obtain an overview of the extract composition. A total of 18 compounds, including l-Tyrosine, 5-Hydroxymethyl-2-furaldehyde, Citraconic acid, Kojic acid, D-(+)-Tryptophan, DHQ, Genistein, Quercetin, 7-hydroxy-6-methoxy-2H-chromen-2-one, Emodin, Daidzein, Epimedin B, Icariin, 7-hydroxy-3-(4-methoxyphenyl)-4H-chromen-4-one, Octadecanamine, Andrographolide, Eucalyptol, and Astragaloside A, were identified. DHQ was also determined in HPLC: its content was quantified by comparing its peak area with that of the certified reference standard of known concentration. RAW264.7 cells, previously stimulated with LPS (1 µg/mL), were treated with PPE concentrations ranging from 20 to 140 µg/mL. Cell Counting Kit-8 (CCK-8) assay revealed that none of the tested concentrations affected cell viability; on the contrary, induction of proliferation was observed. PPE, at the concentration of 80 µg/mL, inhibited NO production and the release of IL-17, TNF-α, IL-6 and IL-1β. Western blot analyses highlighted that PPE suppressed NF-kB activation, JAK1 and STAT3 expression. The PPE anti-psoriatic properties were also investigated in BALB/c mice, treated with PPE low (50 mg/kg), medium (100 mg/kg) and high (200 mg/kg) doses, per oral administration (7 days). Skin lesions (scaling, epidermal surface) and splenomegaly appeared significantly ameliorated after PPE treatment. In particular, the dose of 200 mg/kg showed a resolution compared to that obtained through methotrexate (4 mg/kg) [47].

### 2.15. Centipeda minima (L.) A.Braun & Asch.

Ma and colleagues (2023) [48] investigated the potential anti-psoriatic effects of *Centipeda minima* (L.) A.Braun & Asch. extract, enriched with Brevilin A, Arnicolide D, Arnicolide C and Microhelenin Cin macrophage and keratinocyte models. The extract inhibited mRNA expression of IL-6 and TNF-α stronger than Arnicolide D and suppressed CCL20 production more than Arnicolide C in LPS-stimulated RAW264.7 cells. Moreover, the extract downregulated JAK1, JAK2, STAT1 and STAT3 phosphorylated proteins. In keratinocyte models, it was observed that the extract significantly inhibited both IL-6 induced phosphorylation of JAK1, JAK2, and STAT3 proteins and the IFN-γ-induced release of CCL17, CXCL9, CXCL10 chemokines. All these findings suggest that the extract, thanks to the anti-inflammatory and antiproliferative properties it exerts, could be considered a potential therapeutic agent for psoriasis [48].

**Table 1:** Plant extracts downregulating the JAK/STAT Signaling Pathway in in vitro and in vivo induced psoriatic models.

Plant Species	Model	Tested Concentrations/ Doses	JAK/STAT Downregulation	Pathways and Cytokines Downregulation	References
*F. carica*	RAW264.7 cells	from 50 to 200 µg/mL	p-JAK1 p-STAT3	NO, iNOS	[35]
	BALB/c mice	10 mg/mL	-	-	
*C. asiatica*	HaCaT cells	25 µg/mL	p-JAK1 p-STAT3	CCL20, IL-6, TNF-α, AQP3, FLG, TLR4/NF-kB	[36]
	IMQ 5% induced psoriasis-like mice	40 mg/mL	-	-	
*A. dioicus* var. *kamtschaticus*	HaCaT cells	50, 100, 200 µg/mL	p-JAK1 p-STAT3	CXCL1, CXCL2, CXCL10, CCL20, S100A8, S100A9, IL-1β, IL-23	[37]
	IMQ-induced psoriasis-like BALB/c mice	1 mg/mouse, 2 mg/mouse	-	-	
*G. superba*	HaCaT cells	0.01–10 µg/mL	p-STAT3	K17	[38]
*C. roseus*	HaCaT cells	10, 25, 50, 100 µg/mL	p-STAT3	K17	[38]
*I. verum*	HaCaT cells	25, 50, 100 µg/mL	p-JAK2 p-STAT1	-	[39]
*S. mairei*	HaCaT cells	From 0.025 to 0.05 mg/mL	-	IL-6	[40]
	IMQ-induced psoriasis-like dermatitis mice	150 mg/kg, 300 mg/kg	p-JAK2 p-STAT3	NF-kB, IL-1β, IL-6, IL-8, IL-17, IL-22, IL-23	
*Citrus* spp. *Z. bungeanum*	HaCaT cells	50 µg/mL	p-JAK2 p-STAT3	p-38 MAPK, TNF-α, IL-6, IFN-γ, CCL20, MMP1, NF-kB	[41]
*H. dulcis*	HaCaT cells	6.25,25 µg/mL	p-STAT3	IL-1α, IL-1β, CCL-20, CXCL-8, NF-kB	[42]
*Rhizoma Smilacis Glabrae*	IMQ-induced psoriasis BALB/c mice	23.3 mg/kg, 35.0 mg/kg 46.6 mg/kg	JAK/STAT	IL-17, IFN-γ, TNF-α, Rig-1, Gata-3, mTOR, p-65	[43]
	HaCaT cells	-	-	Th17/Treg	
*H. hirta*	HaCaT cells	60, 120, 240 µg/mL	p-JAK2 p-STAT1 p-STAT3	IL-6, CCL22, CCL5, JNK/MAPK	[44]
*T. cordifolia*	Native mouse CD4^+^ T cells	500, 1000, 1500 and 2000 µg/mL	JAK/STAT	TLR-3, TLR-4	[45]
*L. angustifolia*	HaCaT cells	20, 40, 100 µg/mL	p-JAK2 p-STAT1	NF-kB, P13K/Akt, MAPK8, IL-6, CCL20	[46]
		5, 10, 25 µM for Rosmarinic Acid			
*P. massoniana*	RAW264.7 cells	80 µg/mL	p-JAK1 p-STAT3	NO, IL-17, TNF-α, IL-6, IL-1β	[47]
	BALB/c mice	50 mg/kg, 100 mg/kg, 200 mg/kg	-	-	
*C. minima*	RAW264.7 cells	-	p-JAK1 p-STAT2 p-STAT3	IFN-γ, CCL17, CXC19, CXCL10	[48]

## 3. Herbal Remedies Formulas Targeting the JAK/STAT Signaling Pathway in In Vitro and In Vivo Induced Psoriatic Models

**Figure 2:**
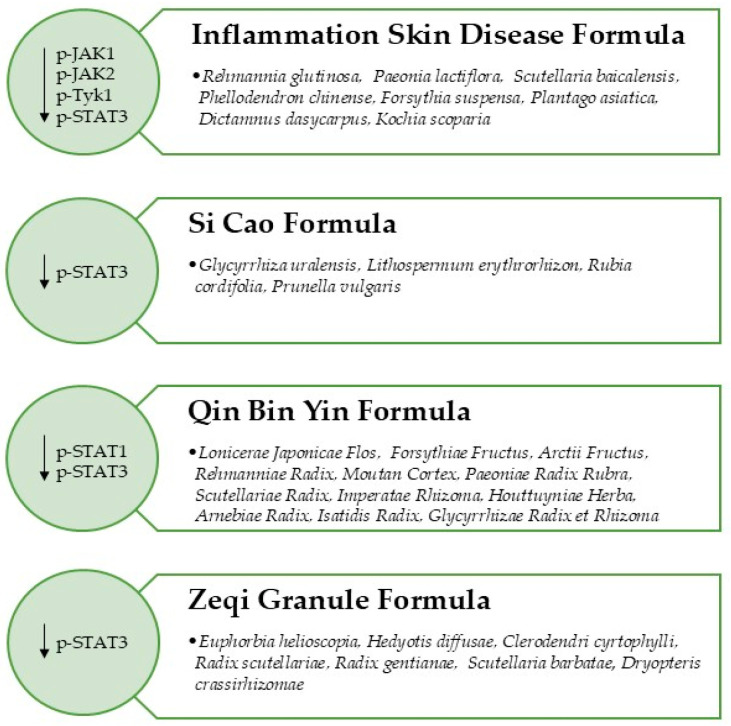
Herbal formulas downregulating the JAK/STAT Signaling Pathway in in vitro and in vivo induced psoriatic model.

Traditional medicine, specifically traditionally used herbs, represent a valuable source of compounds for the treatment of several diseases including psoriasis [49]. Herbal remedy formulas able to modulate the JAK/STAT Signaling Pathway in psoriatic models are discussed below (Figure 2).

### 3.1. Inflammation Skin Disease Formula

Inflammation Skin Disease Formula (ISDF) is a Chinese Herbal Medicine Formula containing nine herbs, including the following: dried roots of *Rehmannia glutinosa*, *Paeonia lactiflora*, *Scutellaria baicalensis*, dried cortex of *Phellodendron chinense*, dried fruits of *Forsythia suspensa*, dried seeds of *Plantago asiatica*, dried root barks of *Dictamnus dasycarpus* and dried ripe seeds of *Kochia scoparia*. Wang and colleagues (2024) [50] investigated the anti-psoriatic effects of ISDF. Raw matrix was extracted in EtOH 60% through ultrasonication; the extract contained baicalin 4.92%, berberine 2.90%, paeoniflorin 0.26% and phillyrin 0.10%. A total of 48 adult BALB/c mice were treated with 3.15 g/kg, 6.30 g/kg and 9.45 g/kg of ISDF, 7 days after the first IMQ sensitization. Forty-eight C57 mice were pre-treated with ISDF and dexamethasone (used as positive control), then stimulated with IL-23 cytokine injection. Data showed that ISDF treatment did not affect mice body weight and IMQ-induced increased thickness of dorsal skin. On the other hand, the overall PASI score for animals treated with 3.15 g/kg, 6.30 g/kg and 9.45 g/kg decreased by 35.16%, 48.54% and 79.41%, respectively. The IL-17A serum levels appeared significantly suppressed, while IL-22 and IL-6, even if with no statistically significant results, demonstrated a decreasing trend. In particular, a 14-day treatment with ISDF (6.30 g/kg and 9.45 g/kg) attenuated markers of inflammation within mice serum and decreased NF-kB, p65 and MAPK pathways. ISDF reduced phosphorylation of JAK1, JAK2, Tyk2 and STAT3 proteins up to 30.70%, 71.42%, 72.60% and 75.34%, respectively, showing involvement in modulating the JAK/STAT Signaling Pathway [50].

### 3.2. Si Cao Formula

Si Cao is an ancient formula containing 50 g of *Glycyrrhiza uralensis* roots and rhizomes, 10 g of *Lithospermum erythrorhizon* roots, 10 g of *Rubia cordifolia* roots and 10 g of *Prunella vulgaris* ear of grain. The herb was subjected to extraction in EtOH 80% and implemented with sunflower seed oil. Liquiritin and oxymatrine were the main identified compounds at HPLC analysis. A randomized, controlled, non-blinded pilot clinical study was conducted on 30 participants with diagnosis of mild-to-moderate plaque psoriasis, treated randomly with calcipotriol ointment and Si Cao formulation for 8 weeks. Also, 8 BALB/c mice, stimulated with IMQ, were treated with Si Cao formula. For the clinical study, the investigated PASI score result was similar to that of the control group after 8 weeks of treatment. Skin lesions were significantly attenuated while area shrank and plaque results decreased. Even at follow-up, a drastically decreased recurrence rate (33.3%) was observed. Si Cao application also reduced epidermal proliferation and angiogenesis and ameliorated skin lesion in IMQ-treated mice. Furthermore, decreased mRNA expression levels of both IL-17A and STAT phosphorylated proteins, as well as reduced T cell infiltration in the epidermis, were observed in mice [51].

### 3.3. Zeqi Granule

Zeqi Granule is composed of seven herbs: *Euphorbia helioscopia*, *Hedyotis diffusae*, *Clerodendri cyrtophylli*, *Radix scutellariae*, *Radix gentianae*, *Scutellaria barbatae*, and *Dryopteris crassirhizomae*. Wu and colleagues (2024) [52] investigated the anti-psoriatic effects of the Zeqi decoction in animals. BALB/c mice were randomly treated with 29.25 g/kg/d of the formulation for 7 days. PASI score was evaluated: erythema, scales, thickness, and infiltration degree of skin lesion were significantly reduced. mRNA expression of STAT1 and STAT3 proteins appeared reduced in the treatment group, while the expression level of IL-10 anti-inflammatory cytokine was induced [52].

### 3.4. Qin Bi Yin

Qin Bi Yin decoction derives from Traditional Chinese Medicine. It is prepared by mixing 15 g of Lonicerae Japonicae Flos, 15 g of Forsythiae Fructus, 15 g of Arctii Fructus, 30 g of Rehmanniae Radix, 12 g of Moutan Cortex, 12 g of Paeoniae Radix Rubra, 10 g of Scutellariae Radix, 30 g of Imperatae Rhizoma, 20 g of Houttuyniae Herba, 12 g of Arnebiae Radix, 10 g of Isatidis Radix, and 10 g of Glycyrrhizae Radix and Rhizoma. The most abundant bioactive compounds, quercetin, baicalein, glycyrrhizic acid, glabridin, forsythin and licoflavone A, were assessed through Ultra High-Performance Liquid Chromatography-Q Exactive Orbitrap-Mass Spectrometry (UHPLC-QE-MS). The use of this advanced analytical technique allowed the overall investigation of Qin Bi Yin’s phytochemical profile. CCK8 assay demonstrated that Qin Bi Yin could inhibit differentiation and proliferation of CD4+ Th17 cells by modulating the S1P/S1PR1 Signaling Pathway. while the ELISA test demonstrated that the formula suppressed both IL-17 and IL-23 in a statistically significant manner. Western blot analyses revealed that Qin Bi Yin treatment increased phosphorylation of SMAD2 and downregulated phosphorylation of STAT3 proteins [53].

## 4. Discussion

### 4.1. Present Insights in Innovative Therapeutic Approaches for Psoriasis Management and Current Findings About Plant Extracts Potential Use

Psoriasis, affecting about 29.5 million adults worldwide, is a chronic, recalcitrant and disabling dermatological condition associated with a systemic inflammatory response. It was traditionally treated with topical agent application and phototherapy, two strategies that alleviate the symptoms but do not affect the cause. Currently, the observation of other immune-mediated inflammatory diseases revealed that early therapy with biologics could be useful both in the treatment and in the follow-up of psoriasis [54]. Biologics, in fact, introduced around 20 years ago, marked a real turning point in psoriasis management [11,55]. A retrospective study, conducted by Sutaria and Au (2019) [56], evaluated the failure rate and the survival times in 250 patients affected by psoriasis. Data showed that systemic therapies had a higher failure rate than biologic ones because of the increased incidence of adverse effects [56]. On the other hand, anti-drug antibody formation against biologics represents a fearsome reason for therapeutic failure, too. Among a population of 42,280 individuals, only 11 biologics, including Secukinumab, Etanercept, and Brodalumab, induced a lower anti-drug antibody formation [57]. At present, the recent introduction of JAK inhibitors, a new and promising small-molecule class of drugs inhibiting the JAK/STAT Signaling Pathway, could represent a turning point in the management of psoriasis [58]. Considering the promising use of these drugs in overall ISD management, this review aimed at investigating the potential role of plant-derived extracts and herbal formulas in JAK/STAT Signaling Pathway inhibition for psoriasis management. The mechanism of action underlying the activity of all the evaluated studies mainly recognizes the inhibition of the JAK/STAT cascade as the common denominator. The in vitro activity of the evaluated extracts was assessed in human keratinocytes HaCaT cells and in murine macrophages RAW264.7 cells, previously induced with pro-inflammatory stimuli. *C. asiatica* [36] and *A. dioicus* var. *kamtschaticus* [37] extracts inhibited phosphorylation of both JAK1 and STAT3 phosphorylated proteins in HaCaT cells, while *F. carica* [35], *P. massoniana* [47] and *C. minima* [48] extracts induced the same effects in LPS-stimulated RAW264.7 cells. *G. superba* and *C. roseus* downregulated STAT3 phosphorylation and suppressed K17 expression in IFN-γ stimulated HaCaT cells [38]. Besides the JAK/STAT Signaling Pathway inhibition, *Citrus* spp. samples, *Z. bungeanum* [41] and *H. hirta* [44] downregulated the NF-kB pathway and decreased IL-6 production, both factors which are well recognized for their involvement in inflammatory conditions. For instance, all the effects were observed at concentrations that did not affect cell viability. In vivo studies, mainly conducted in psoriasis-like dermatitis mice treated with IMQ, demonstrated that both *S. mairei* [40] and *Rhizoma Smilacis Glabrae* [43] extracts inhibited the JAK/STAT Signaling Pathway and downregulated IL-17 cytokine levels. An overview of the cited studies highlighted that decreased levels of IL-6 were observed in most of the works, directly suggesting the key role of this pro-inflammatory cytokine in the psoriasis process. Among the discussed herbal formulas, the efficacy of the Si Cao Formula, verified through a pilot clinical study, stands out. Data revealed beneficial effects both at the end of the treatment and at follow-up [51]. A critical analysis of the studies conducted on IMQ-stimulated mice treated with all the other investigated formulations indicated that the activity was mediated by p-STAT protein inhibition [50,52,53].

### 4.2. Concluding Remarks: Gaps in the Literature, Safety Concerns and Future Perspective

The use of vegetable substances did not show frustration, mood swings, diarrhea or vomiting, all symptoms typical of patients affected by psoriasis under pharmacological treatments [59]. According to Mustafa and colleagues (2025), by exerting a plethora of biological activities, from antioxidant to anti-inflammatory and from immunomodulatory to antiproliferative, plant species can act as key players able to induce multi-target effects [60]. A critical analysis of the screened publications revealed a literature gap in both clinical translation and clinical validation of plant extracts’ therapeutic efficacy in dermatological conditions. Most of the current literature, in fact, with the only exception of a pilot study, is limited to in vitro and in vivo studies. Even if the availability, low costs and efficacy of medicinal plants are known, more in vitro and in vivo studies with proper experimental designs and standard protocols and evaluating efficacy and safety-related issues are needed [61,62,63]. Furthermore, since medicinal plants can cause intoxication, nephritis, liver damage and, in some cases, abortion, toxicological evaluations and long-term use studies are necessary [64]. Another issue that needs to be addressed concerns the clear limitations in both application and approval processes of plant-derived extracts [65]. Overall reliability and predictability of plant extracts’ therapeutic effects can be assessed by evaluating properties of standardized products only [66]. The most evident obstacles for translation to clinical use, in fact, are the lack of extract standardization, the use of small sample size and the occurring limiting pharmacokinetic parameters. The development of innovative formulation technologies could partially overcome this last issue [60,67,68,69,70]. The type of formulation used, in fact, needs to be considered, as it could substantially affect the therapeutic potential activity of plant-based products. Traditional formulations such as creams, ointments, gels and lotions showed only acceptable or poor therapeutic potential linked to the loss of ceramide and barrier function affecting psoriatic skin. On the contrary, new drug delivery systems, including liposomes, lipospheres, nanostructured lipid carriers, crystals, spheres niosomes, ethosomes, microneedles and foams, are designed to improve penetrability and, consequently, to ameliorate hydration power [34]. Furthermore, another strategy that deserves more attention concerns the use of plant extracts alongside pharmacological therapy for psoriasis: this could allow low drug dosage and, consequently, mitigate side effects [69].

## 5. Methods

For this study, a comprehensive search, performed throughout the major bibliographic databases, including Google Scholar, PubMed and Scopus, was conducted between December 2025 and January 2026. The following keywords of “Plant Extracts”, “Psoriasis”, “JAK/STAT”, “JAK/STAT Signaling Pathway” were matched, and Boolean operators (“AND”, “+” “OR”) were introduced in between in order to maximize retrieval. Duplicate papers found in the different databases were removed. An initial major screening was conducted by evaluating material by title and abstract; then, full-text evaluation was performed. Selected articles had to match the following inclusion criteria: be published in peer-reviewed journals and be written in English. Review articles, conference abstracts, editorials, proceeding papers and book chapters were excluded. Out-of-scope publications, not related to psoriasis, not related to JAK/STAT Signaling Pathway, and not related to plants species, were excluded.

## 6. Conclusions

It is widely recognized that dysregulation of the JAK/STAT Signaling Pathway is associated with several different diseases, including inflammatory and immune-mediated disorders. A growing body of evidence proposes the JAK/STAT Signaling Pathway as a potential therapeutic target in psoriasis management, since it can be hit at different levels and through different strategies [24]. Plant species, due to their well-known multi-target effects, can be considered interesting candidates in attenuating JAK/STAT Signaling Pathway activation both in in vitro and in vivo psoriatic models. The emerging data reveal potential that deserves further investigation.

## Data Availability

No new data were created or analyzed in this study. Data sharing is not applicable to this article.

## Abbreviations

The following abbreviations are used in this manuscript:

Akt/mTOR	Protein Kinase B/Mammalian Target of Rapamycin
AMPK	AMP-Activated Protein Kinase
AQP3	Aquaporin 3
CCK-8	Cell Counting Kit-8
CD3^+^ T cells	Cluster of Differentiation 3-positive T Cells
CCL20	C-C Motif Chemokine Ligand 20
COVID-19	Coronavirus Disease 2019
CXCL	C-X-C Motif Chemokine Ligand
EMA	European Medicine Agency
FDA	Food and Drug Administration
FLG	Filaggrin
Gata3	GATA binding protein 3
GSH	Glutathione
HPLC	High-Performance Liquid Chromatography
ICAM1	Intercellular Adhesion Molecule 1
IFN-γ	Interferon-gamma
IL	Interleukin
IMQ	Imiquimod
iNOS	Inducible Nitric Oxide Synthase
ISD	Inflammatory Skin Diseases
JAK/STAT	Janus Kinase Signal Transducer and Activator of Transcription
JNK	c-jun N-terminal kinase
K17	Keratin 17
LPS	Lipopolysaccharide
MAPK	Mitogen-Activated Protein Kinase
MMP1	Matrix Metalloproteinase-1
NF-kB	Nuclear Factor-kappa B
NO	Nitric Oxide
PASI	Psoriasis Area and Severity Index
PRISMA	Preferred Reporting Items for Systematic Reviews and Meta-Analyses
Rig-1	Retinoic acid-inducible gene I
ROS	Reactive Oxygen Species
S100A8	S100 calcium binding protein A8
S100A9	S100 calcium binding protein A9
SOCS	Suppressor of Cytokine Signaling
SOD	Superoxide Dismutase
Th17	T helper 17 cells
TLR4/NF-kB	Toll-Like Receptor 4/Nuclear Factor-kappa B
TNF-α	Tumor Necrosis Factor-alpha
UPLC-Q-Orbitrap/MS	Ultra High-Performance Liquid Chromatography-Quadrupole-Orbitrap Mass Spectrometry
VEGF	Vascular Endothelial Growth Factor
